# Wearable sensor-based gait analysis to discriminate early Parkinson’s disease from essential tremor

**DOI:** 10.1007/s00415-023-11577-6

**Published:** 2023-02-01

**Authors:** Shinuan Lin, Chao Gao, Hongxia Li, Pei Huang, Yun Ling, Zhonglue Chen, Kang Ren, Shengdi Chen

**Affiliations:** 1 GYENNO SCIENCE CO., LTD., Shenzhen, 518000 China; 2 Department of Neurology, Ruijin Hospital, Shanghai Jiao Tong University School of Medicine, 197 Ruijin Er Road, Shanghai, 200025 China; 3 HUST–GYENNO CNS Intelligent Digital Medicine Technology Center, Wuhan, 430074 China

**Keywords:** Parkinson’s disease, Essential tremor, Wearable sensor, Gait, Machine learning

## Abstract

**Background:**

Differentiating early-stage Parkinson's disease (PD) from essential tremor (ET) is challenging since they have some overlapping clinical features. Since early-stage PD may present with slight gait impairment and ET generally does not, gait analysis could be used to differentiate PD from ET using machine learning.

**Objective:**

To differentiate early-stage PD from ET via machine learning using gait and postural transition parameters calculated using the raw kinematic signal captured from inertial measurement unit (IMU) sensors.

**Methods:**

Gait and postural transition parameters were collected from 84 early-stage PD and 80 ET subjects during the Time Up and Go (TUG) test. We randomly split our data into training and test data. Within the training data, we separated the TUG test into four components: standing, straight walk, turning, and sitting to build weighted average ensemble classification models. The four components’ weight indices were trained using logistic regression. Several ensemble models’ leave-one-out cross-validation (LOOCV) performances were compared. Independent test data were used to evaluate the model with the best LOOCV performance.

**Results:**

The best weighted average ensemble classification model LOOCV results included an accuracy of 84%, Kappa of 0.68, sensitivity of 85.9%, specificity of 82.1%, and AUC of 0.912. Thirty-three gait and postural transition parameters, such as ***Arm–Symbolic Symmetry Index*** and ***180° Turn–Max Angular Velocity***, were included in Feature Group III. The independent test data achieved a 75.8% accuracy.

**Conclusions:**

Our findings suggest that gait and postural transition parameters obtained from wearable sensors combined with machine learning had the potential to distinguish between early-stage PD and ET.

**Supplementary Information:**

The online version contains supplementary material available at 10.1007/s00415-023-11577-6.

## Introduction

Essential tremor (ET) and Parkinson's disease (PD) are two common movement disorders in older populations [1–3] and have some overlapping clinical features, e.g., rest and postural tremors, making it difficult to differentiate ET and PD in their early stages [4–7].

Early-stage PD and ET can be distinguished by the following clinical symptoms: (1) Tremor features: rest tremor is usually an early sign in PD, while action tremor is usually an early sign of ET, and rest tremor may be present years after disease onset in ET. Re-emergent tremor can be present in PD but absent in ET. (2) Bradykinesia: bradykinesia is the prerequisite for PD diagnosis, and bradykinesia in patients with PD often manifests as poor hand flexibility in the early stages. However, ET is not usually associated with bradykinesia. (3) Rigidity: PD is associated with rigidity, whereas ET usually is not. (4) Gait: early-stage PD may manifest as reduced stride length, slow speed, poor symmetry, and reduced amplitude of arm swing on the affected side. However, gait disorder is usually not present in ET [8].

However, despite the differences in the above symptoms between PD and ET, it is still challenging to distinguish early-stage PD from ET. First, rest tremor, postural tremor, or action tremor can be present in both ET and PD patients at the early stage. Second, some PD patients are only with tremor and other motor symptoms are very mild or not obvious, which are difficult to be detected by subjective observation and physical examination. Therefore, in recent years, some researchers have begun to use devices to quantitatively identify the above-mentioned motor symptoms, such as bradykinesia, tremor, and gait, hoping to achieve early diagnosis of PD.

In previous studies, researchers have mainly focused on the characteristics of the patient’s tremors to differentiate the diseases [9–12]. Long-term EMG recordings with/without combined accelerometers were used to differentiate the two disorders [9, 11]. EMG analysis might help differentiate the two disorders, but it is an invasive examination and is limited for application when patients have mixed types of tremors.

Gait impairment and bradykinesia have been reported in early-stage PD [13]. However, it is not easy to distinguish the gait impairment of early-stage PD from ET by subjective assessment, since these symptoms are minor. Therefore, advanced technologies, such as wearable sensors and motion capture systems, have been used for clinical differentiation of the diseases according to gait and balance parameters [14, 15]. Moon et al. [14] obtained some gait and balance characteristics from inertial measurement unit (IMU) sensors during the instrumented stand and walk test to discriminate PD and ET (average durations (years), PD: 8.2, ET: 13.83). The results showed that the cross-validation F1 score was 0.61 for the best model. However, it was not clear if this classification system could be applied to differentiate early-stage PD from ET. The Time Up and Go (TUG) test has been widely used as an assessment for gait and balance problems in movement disorders, including PD and ET [16, 17], and its use in combination with IMU sensors was recommended for capturing the raw kinematic signal to quantitatively analyze the gait [18, 19].

Segmentation of the TUG test into phases provides additional parameters [20] that might be helpful for differentiating PD from ET. Therefore, we separated the entire TUG test into four components (standing, straight walk, turning, and sitting) and integrated them into a final weighted average ensemble classification model. In our diagnostic study, we primarily examined whether wearable sensor-based gait and postural transition parameters obtained from the TUG test could be used as input features in machine learning algorithms to differentiate early-stage PD from ET.

## Materials and methods

### Participants

This study was approved by the Ethics Committee of Ruijin Hospital, Shanghai Jiao Tong University School of Medicine. Written informed consent was obtained from all the participants. Eighty-four subjects with PD (age: 58.13 ± 10.43) and eighty age-matched ET subjects (age: 58.7 ± 13.9) participated in this study at Ruijin Hospital, Shanghai Jiao Tong University School of Medicine between October 2019 and November 2021. PD and ET subjects were diagnosed by two movement disorder specialists according to the Movement Disorder Society (MDS) PD criteria [21] and ET criteria [22]. Only early-stage PD [Hoehn and Yahr (H&Y) stage 1–1.5)] [23] and ET patients who had limb tremor symptoms were recruited into this study. The exclusion criteria were as follows: (1) a history of cerebrovascular disease (e.g., infarction, hemorrhage), brain tumor, head trauma or any psychiatric disorders; (2) a history of medication known to cause parkinsonism or affect clinical assessment; (3) orthopedic impairment or other disease that likely contributed significantly to gait disturbance; (4) MMSE < 24 or cognitive disorder that likely contributed significantly to gait disturbance; and (5) participants who had both PD and ET. The demographic data and clinical characteristics of participants are provided in Table 1.

**Table 1:** Demographic characteristics of the early-stage PD subjects and ET subjects

	Entire dataset			Training data			Test data		
	ET (*n = *80)	PD (*n = *84)	*p* value^a^	ET (*n = *67)	PD (*n = *64)	*p* value^a^	ET (*n = *13)	PD (*n = *20)	*p* value^a^
Age, y	58.70 (13.90)	58.13 (10.43)	0.767	58.57 (14.04)	57.06 (10.80)	0.494	59.38 (13.71)	61.55 (8.54)	0.579
Sex: Female no. (%)	43 (53.8)	38 (45.2)	0.351	37 (55.2)	29 (45.3)	0.337	6 (46.2)	9 (45.0)	1
Height, cm	164.12 (18.78)	165.75 (8.08)	0.469	165.90 (8.28)	165.94 (8.13)	0.977	155.00 (42.86)	165.15 (8.11)	0.307
Education: no. (%)			0.029			0.155			0.01
No formal qualification	4 (5.0)	3 (3.6)		3 (4.5)	1 (1.6)		1 (7.7)	2 (10.0)	
Primary school	11 (13.8)	10 (11.9)		11 (16.4)	8 (12.5)		0 (0.0)	2 (10.0)	
Junior high school	10 (12.5)	30 (35.7)		8 (11.9)	20 (31.2)		2 (15.4)	10 (50.0)	
Senior high school	23 (28.7)	15 (17.9)		17 (25.4)	15 (23.4)		6 (46.2)	0 (0.0)	
College/bachelor’s degree	29 (36.2)	24 (28.6)		25 (37.3)	18 (28.1)		4 (30.8)	6 (30.0)	
Advanced degree	3 (3.8)	2 (2.4)		3 (4.5)	2 (3.1)		0 (0)	0 (0)	
Disease duration, y	9.65 (9.36)	4.56 (4.99)	< 0.001	9.67 (9.80)	4.77 (5.30)	0.001	9.56 (7.09)	3.91 (3.87)	0.006
H&Y: no. (%)	–		–	–		–	–		–
1		39 (46.4)			31 (48.4)			8 (40.0)	
1.5		45 (53.6)			33 (51.6)			12 (60.0)	
MDS-UPDRS III	–	21.39 (12.49)	–	–	21.95 (13.32)	–	–	19.60 (9.44)	–
Taking dopaminergic therapy: no. (%)	–	39 (46.4)	–	–	34 (53.1)	–	–	5 (25.0)	–

Data are shown as the mean (SD) for continuous variables and *n* (%) for categorical variables

*ET* essential tremor; *PD* Parkinson’s disease; *MDS-UPDRS* MDS-Unified Parkinson's Disease Rating Scale

^a^*p* value: Differences between groups were assessed using the chi-square test for categorical variables and two-sample t test (two-sided) for continuous variables

### Protocol and materials

**Fig. 1:**
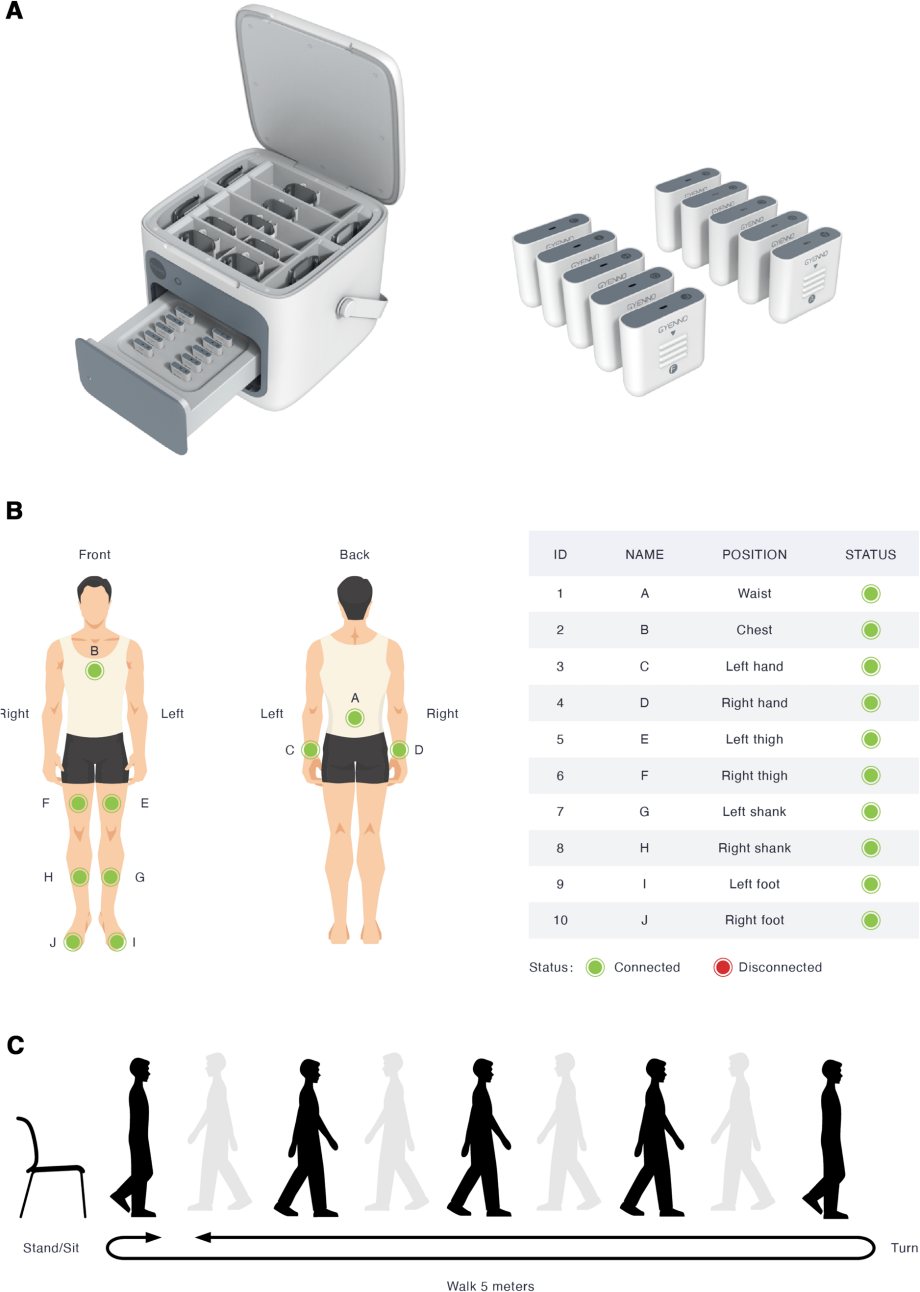
**A** Sensor overview. **B** Sensor locations. **C** TUG test procedure

A wearable motion and gait quantification assessment system, MATRIX (GYENNO SCIENCE, Shenzhen, China), which is commercially available, was utilized in this study [24]. It is approved by the National Medical Products Administration (NMPA), U.S. Food and Drug Administration (FDA), and Conformitè Europëenne Medical (CE Medical). All participants were equipped with 10 IMU sensors, with a sampling rate of 100 Hz (Fig. 1A). Each IMU provided inertial sensing results via a (1) tri-axial accelerometer (range = ± 16 g, sensitivity = 16,384 LSB/g) and a (2) tri-axial gyroscope (range =  ± 2000 dps, sensitivity = 131 LSB/dps). Two hand sensors were bilaterally placed on the dorsal side of the wrist. The chest sensor was placed on the sternum of the chest, and the waist sensor was attached to the fifth lumbar vertebra. Two thigh sensors were bilaterally placed 7 cm above the knee, while two shank sensors were bilaterally placed 7 cm below the knee. Two-foot sensors were bilaterally placed at the instep (dorsal side of the metatarsus) of each foot. All sensors were tightened to designated locations by straps (Fig. 1B). The TUG test was performed (Fig. 1C). During the TUG test, participants were instructed to stand up from a chair, walk in a straight line for 5 m at a comfortable speed, turn 180° around at the end of the 5 m marker, walk back to the start point, turn 180° around in front of the chair and sit down on the chair. The raw kinematical signals of participants during TUG tests were captured using the ten wearable sensors in real time and were transmitted to the host computer via a Bluetooth link for further analysis. A total of 184 gait and postural transition parameters (Appendix A, Table 4) based on the raw kinematical signals were calculated automatically by our prebuilt MATLAB algorithm [24]. An introduction of the gait cycles and segmentation of the TUG test is shown in Appendix C.

### Data analysis

#### Data were split into a training dataset and testing dataset

**Fig. 2:**
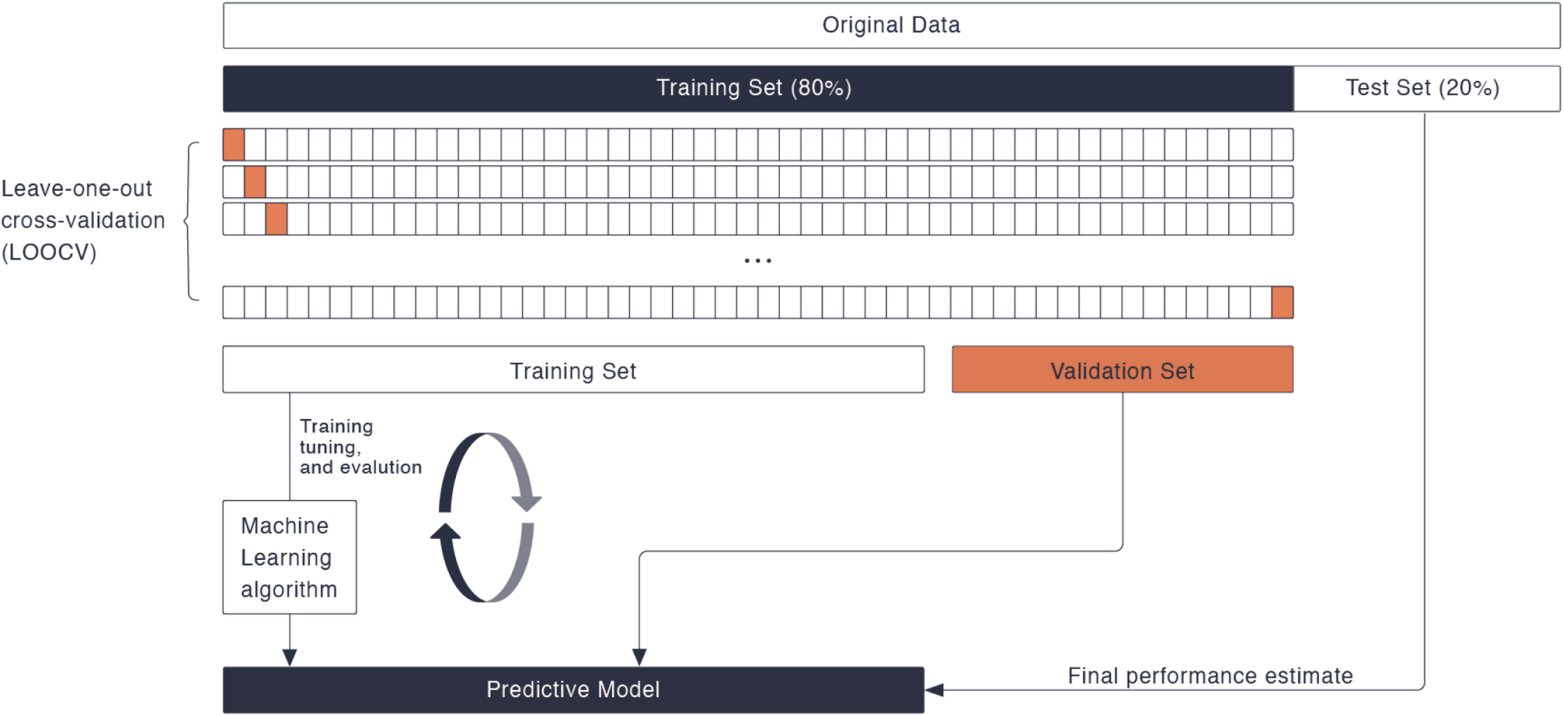
Model training and independent test data evaluation

The entire dataset included 164 recordings (PD: 84, ET: 80), of which 80% of the recordings (PD: 67, ET: 64) were used for training, whereas the remaining 20% (ET: 13, PD: 20) were used for independent testing. In the training dataset, we ensured that the age, sex, and height between the PD and ET groups were matched. Leave-one-out cross-validation (LOOCV) was performed to fine-tune the model parameters in the training data. The final model was selected among different candidate models based on model LOOCV performance. Independent testing was used to provide an unbiased evaluation of the final model (Fig. 2).

### Weighted average ensemble classification model construction

Several feature selection methods were tried (Appendix D) and generated three different feature groups, FG I, FG II and FG III. We trained support vector machine (SVM) and random forest (RF) models on the training subset with features from different feature groups. For each feature group, we trained the models for four components, straight walk, sitting, standing and turning, with corresponding component features separately using LOOCV and obtained 4 probabilities of having PD for each subject. These probabilities were used as input variables for building logistic regression models. The logistic regression coefficients were combined into weights used in a linear combination of the previous 4 probabilities, resulting in an ensemble learning prediction probability score ($P\_{\text{val}}$) for each subject in the training set. If $P\_{\text{val}}$ was > 0.5, the subject would be classified as having PD using our model; otherwise, they would be classified as having ET. This kind of model was called the weighted average ensemble classification model. We selected the weighted average ensemble classification model, which had the best model performance among all models, as our final model. Independent test data were used to evaluate the final model. We calculated the ensemble learning prediction probability score of each subject in the test data $P$ by multiplying the weights obtained during the above training process with the test data predicted scores obtained from four different component models. If *P* was > 0.5, the subject in the test data would be classified as having PD using our model, otherwise they would be classified as having ET (details about ensemble classification model construction are in Appendix E).

### Performance evaluation

The classification models were evaluated with accuracy, kappa, sensitivity, specificity, and AUC. An ROC curve is a graph showing the classification model performance at all different classification thresholds. AUC is the area under the ROC curve. In our case, we set PD as a positive case and ET as a negative case. The accuracy, sensitivity, specificity, and kappa [25] were calculated as follows (T*P = *true positive, TN* = *true negative, F*P = *false positive, FN* = *false negative):

$${\text{Accuracy}} = \frac{{{\text{TP}} + {\text{TN}}}}{{{\text{TP}} + {\text{TN}} + {\text{FP}} + {\text{FN}}}}$$

$${\text{Kappa}} = \frac{{2 \times ({\text{TP}} \times {\text{TN}} - {\text{FN}} \times {\text{FP}})}}{{({\text{TP}} + {\text{FP}}) \times ({\text{FP}} + {\text{TN}}) + ({\text{TP}} + {\text{FN}}) \times ({\text{FN}} + {\text{TN}})}}$$

$${\text{Sensitivity}} = \frac{{{\text{TP}}}}{{{\text{TP}} + {\text{FN}}}}$$

$${\text{Specificity}} = \frac{{{\text{TN}}}}{{{\text{TN}} + {\text{FP}}}}$$

## Results

### Feature comparisons of gait and transition parameters between early-stage PD and ET

**Fig. 3:**
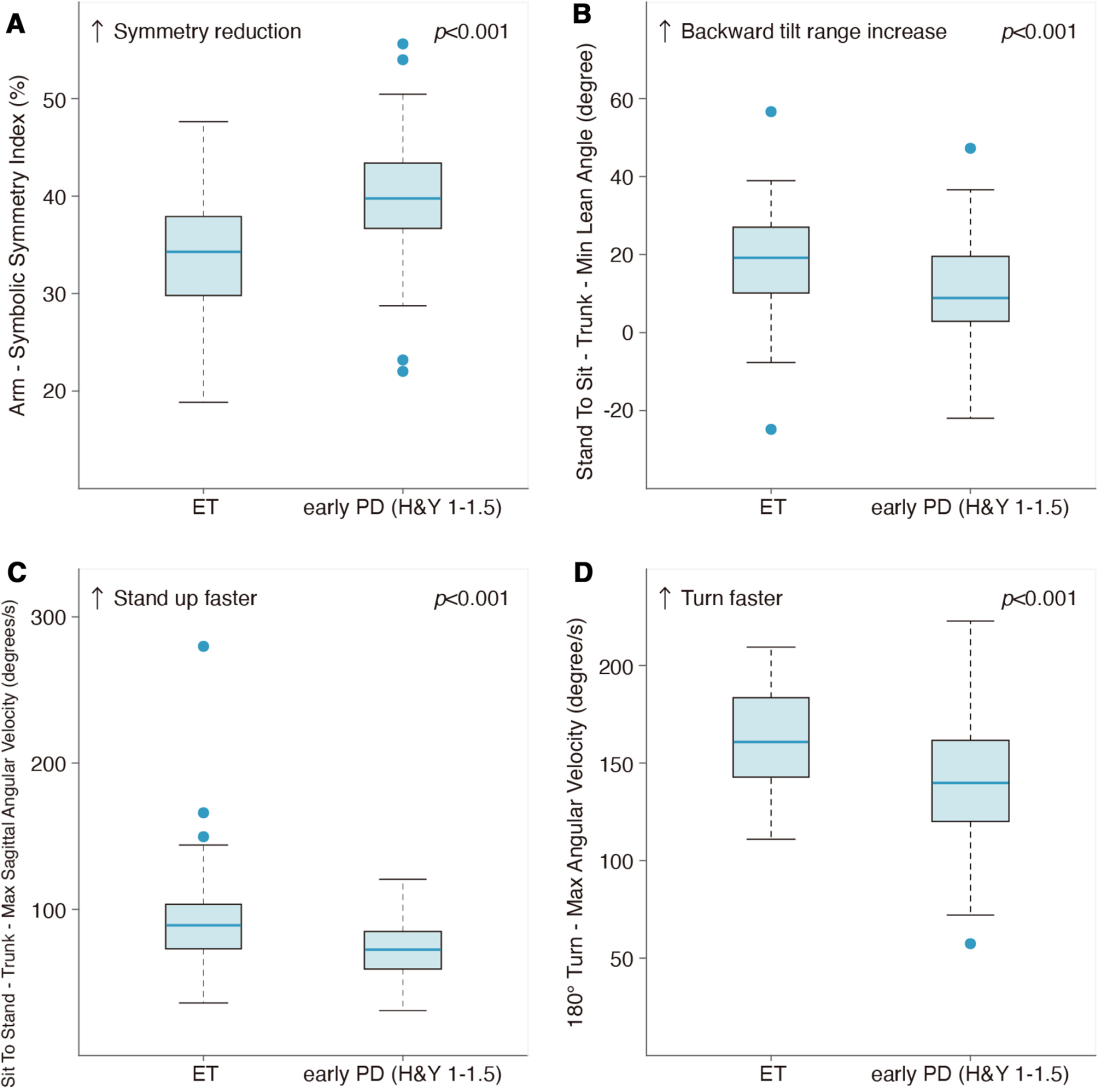
Representative feature comparison between early-stage PD and ET. The box plot represents the following data: the central line represents the median, the top and bottom line of the box represents the 75th quantile (*Q*3) and 25th quantile (*Q*1), the top and bottom of the error bar indicates the “Maximum” (*Q*3 + 1.5 × (*Q*3 − *Q*1)) and “Minimum” (*Q*1 − 1.5 × (*Q*3 − Q1)), dots represent outliers (outside the “Maximum” and “Minimum”). *p*: *P* value was estimated using the Mann‒Whitney U test for exploring feature discrimination ability between the PD and ET groups

Sixty-six out of 214 features were significantly different between early PD and ET (Table 2). Importantly, we found that some feature parameters that differed between early PD and ET were consistent with clinical observations. For example, the ***Arm–Symbolic Symmetry Index*** was higher in PD than in ET by approximately 16.0%. This parameter was used to describe the symmetry of the arms’ movements during the TUG test. The lower the parameter is, the better the symmetry of the arms is. Our results showed that ***Arm–Symbolic Symmetry Index*** was higher in PD than in ET, indicating that PD presents with worse arm symmetry than ET. This finding was consistent with the clinical cases in which early PD usually reduces the arm swing range on the affected side, resulting in bilateral asymmetry, while ET arm swing was not affected. ***Stand To Sit–Trunk–Min Lean Angle*** was lower in PD than in ET by approximately 5.38%. It was defined as the sagittal projection of the maximum backward tilt angle during the sitting process (backward: positive value, forward: negative value). The higher the parameter was, the larger backward tilt the participant had during the sitting process. Our results showed that ***Stand To Sit–Trunk–Min Lean Angle*** was lower in PD than in ET, indicating that PD has a smaller backward tilt range than ET. This finding was consistent with the clinical cases that PD usually has a smaller range of motion. ***Sit To Stand–Trunk–Max Sagittal Angular Velocity*** was smaller in PD than in ET by approximately 18.9%. This parameter is defined as the absolute value of the sagittal projection of the maximum angular velocity of the trunk during the standing process. The higher the parameter is, the faster the participants stand from the chair. Our results showed that ***Sit To Stand–Trunk–Max Sagittal Angular Velocity*** was smaller in PD than ET, indicating that PD stand slower from the chair during TUG testing compared to ET on average. The ***180° Turn–Max Angular Velocity*** was smaller in PD than in ET by approximately 13.0%. This parameter is defined as the maximum value of angular velocity during the turning process. The higher the value is, the faster the participants turn. Our results showed that the ***180° Turn–Max Angular Velocity*** was smaller in PD than in ET, indicating that PD turned slower than ET on average. These two features, ***Sit To Stand–Trunk–Max Sagittal Angular Velocity*** and ***180° Turn–Max Angular Velocity***, were consistent with the clinical cases in which bradykinesia was the main symptom in patients with PD. Although these parameters differ between early PD and ET, box plots of these four features showed overlaps between early PD and ET (Fig. 3), so the combination of more features was needed to achieve a more accurate discrimination.

**Table 2:**
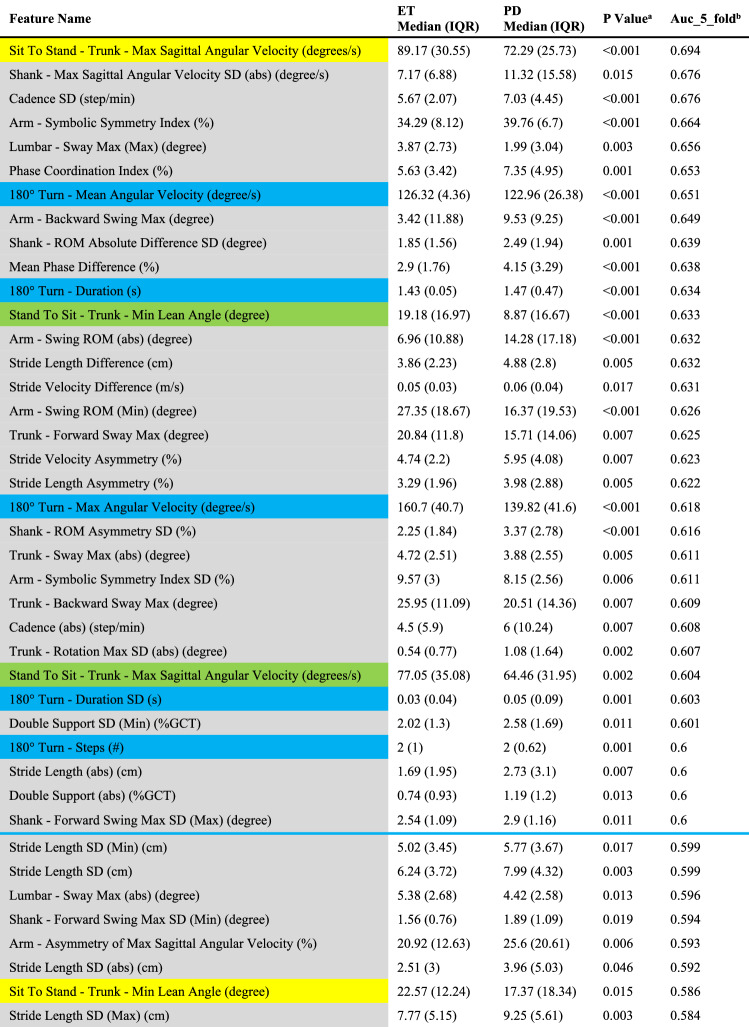

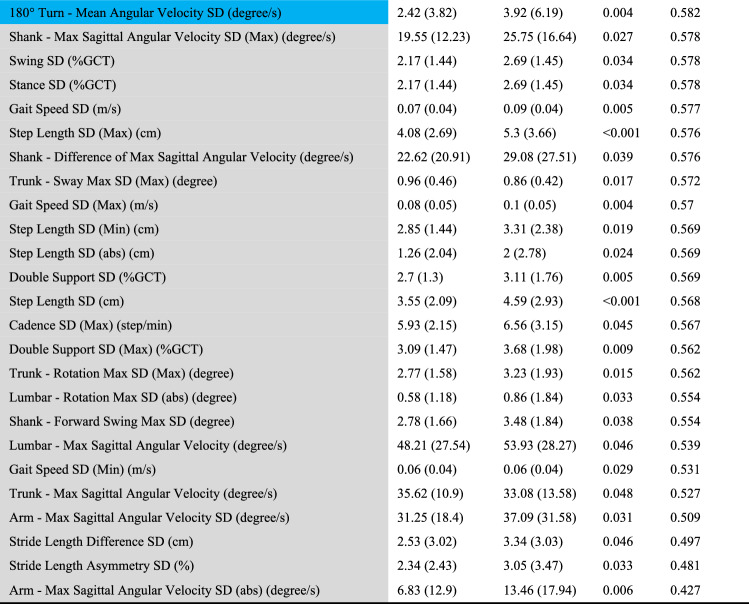
Significant gait and postural transition features obtained from the TUG test

Color coding for features:

Blue line: The features above the blue line had AUC $\ge$ 0.6

*ET* essential tremor, *PD* Parkinson's disease, *GCT* Gait cycle time, *ROM* Range of motion, *Max* maximum value between the pair of left-sided and right-sided parameters, *Min* minimum value between the pair of left-sided and right-sided parameters, *abs* absolute value of the difference between the left-sided parameter and right-sided parameter in the pair

^a^*P* value was estimated using the Mann–Whitney U test for exploring feature discrimination ability between the PD and ET groups

^b^AUC_5-fold: fivefold cross-validation area under the ROC curve; AUC_5_fold for each of the 66 significant features in descending order of AUC_5_fold

### Feature selection

Several feature selection methods were tried in the training data, and the feature combinations were organized into 3 groups based on the feature selection method results.

Method 1: Sixty-six out of 214 features were significantly different between early PD and ET (Table 2); thus, FG I contained 66 features. In addition, 56, 6, 2, and 2 features were obtained from the straight walk, turning, sitting, and standing components, respectively.

Method 2: 34 out of 66 features were kept (eTable 1 in the Online Resource); thus, FG II contained 34 features. In addition, 29, 3, 1, and 1 were obtained from the straight walk, turning, sitting, and standing components, respectively.

Method 3: Out of the 66 significant features, 33 individual features were most discriminative in differentiating early PD from ET, with a fivefold cross-validation ROC AUC ≥ 0.6 (Table 2, above the blue line); thus, FG III contained 33 features. Among these 33 most discriminative features in differentiating early PD from ET, 25 were obtained from the straight walk component, and 5, 2, and 1 were obtained from the turning, sitting, and standing components, respectively.

### Model LOOCV performance comparisons

The segmented model LOOCV performance results and weights of four different components (straight walk, turning, standing, and sitting) in all model (SVM or RF) and feature group (FG I, II, III) combinations are shown in Appendix B Table 5. We found that straight walk achieved the highest weights among the other components in all model and feature group combinations. An example of how to calculate the ensemble learning prediction probability score is shown in Appendix F.

The average ensemble classification model validation performance result was obtained as mentioned in the Methods. The validation results of SVM and RF with FG I, II, and III are shown in Table 3. SVM with FG III outperformed all the other models and feature group combinations [accuracy: 84%, kappa: 0.68, sensitivity: 85.9%, specificity: 82.1%, AUC: 0.912]. Therefore, SVM with FG III was selected as our final weighted average ensemble classification model. Corresponding to this best ensemble model, the validation accuracy of the straight walk component (25 features) for discriminating early PD and ET was 78.6%, which achieved the highest accuracy among all four components since turning (5 features), standing (1 feature), and sitting (2 features) were 67.9%, 76.3% and 61.1%, respectively. Most of the information came from the straight walk component, such as bradykinesia, arm swing range, arm symmetry, etc. This may explain why such a component makes the main contribution to the classification process.

**Table 3:** LOOCV performance of the weighted average ensemble classification models

Model	Feature Group	ACC (%)	Kappa	Sensitivity (%)	Specificity (%)	AUC
**SVM**	FG I	73.3	0.468	84.4	62.7	0.843
	FG II	71.8	0.440	90.6	53.7	0.896
	**FG III**	**84.0**	**0.680**	**85.9**	**82.1**	**0.912**
RF	FG I	73.3	0.465	71.9	74.6	0.818
	FG II	77.1	0.540	70.3	83.6	0.832
	FG III	74.0	0.480	68.8	79.1	0.826

The significance of bold were used to highlight the best performance among all the others in Table 3

LOOCV Accuracy, Kappa, Sensitivity, Specificity and AUC of Support Vector Machine and Random Forest with three different feature groups

### Independent clinical evaluation

In our study, the entire dataset had 164 recordings (PD: 84, ET: 80), in which 80% of the recordings were used for training, whereas the remaining 20% were used for independent testing. The selected final weighted average ensemble classification model (SVM with FG III) was evaluated on these 20% recordings (13 ET, 20 PD). The test data performance of the Weight Average Ensemble Classification Model was as follows: accuracy: 75.8%, kappa: 0.492, sensitivity: 80%, specificity: 69.2%, and AUC: 0.823.

## Discussion

The weighted average ensemble classification model with the basic SVM model and FG III outperformed the other ensemble classification models. Our final ensemble model was evaluated in independent test data and achieved 75.8% accuracy in discriminating between early-stage PD and ET.

### Consistency of discriminative parameters and clinical manifestations

The 33 most classifying gait parameters and postural transition parameters (Table 2, above the blue line), such as ***Arm–Symbolic Symmetry Index***, ***Stand To Sit–Trunk–Min Lean Angle***, ***Sit To Stand–Trunk–Max Sagittal Angular Velocity***, and ***180° Turn–Max Angular Velocity***, were consistent with clinical manifestations. PD patients are characterized by rest tremor, bradykinesia, rigidity and postural instability [26]. They showed speed slow-down and amplitude reduction in turning, arm swing, cadence, and trunk rotation compared with ET [15, 27]. When PD patients were at an early stage, motor symptom asymmetry was especially prominent. Thus, slow velocity of turning/sit/stand, increased arm swing asymmetry, and reduced amplitude of trunk rotation were typical clinical features of early-PD patients [28]. Previously, PD was differentiated from ET simply by physical examination and clinical experience. To our surprise, wearable sensors make it possible to sensitively detect these differential characteristics in early-stage PD patients. Moreover, wearable sensors also provide richer multidimensional information than subjective assessment, which greatly improves the accuracy of differential diagnosis.

### Bradykinesia-related gait parameters correlated with MDS-UPDRS bradykinesia scores

In our study, the bradykinesia scores were calculated based on the MDS-UPDRS motor score according to previous criteria (sum of items 3.4, 3.5, 3.6, 3.7, 3.8, 3.9, and 3.14) [29]. The results showed that several gait parameters that could reflect bradykinesia during gait performance were associated with clinically subjective assessed bradykinesia scores. For example, ***180° Turn–Mean Angular Velocity*** (*r = *-0.44, *p = *0.00027), ***Sit To Stand–Trunk–Max Sagittal Angular Velocity*** (*r = *− 0.41, *p = *0.0008) and ***180° Turn–Max Angular Velocity*** (*r = *− 0.33, *p = *0.008) had a negative correlation with bradykinesia scores, while ***180° Turn–Duration*** had positive correlation (*r = *0.44, *p = *0.00027) with bradykinesia scores. Bradykinesia is the main symptom in PD. Bradykinesia is usually measured according to the UPDRS part III (motor section), but such measurement suffers from low reliability [30, 31]. Interestingly, our study revealed that bradykinesia manifestation could also be presented by our gait analysis system via velocity and time duration parameters. These bradykinesia-related gait parameters could also be used to differentiate between early PD and ET (Table 2, above the blue line).

### The advantage of our study

First, our study separated the entire TUG test into four components and integrated them into a final weighted average ensemble classification model with different weights. The key reason for this separation was considering the different levels of sensitivity to PD/ET for the four components, which should correspond to different weights while building the ensemble classification model. Second, to evaluate our model performance, we kept 20% of our whole dataset as our independent test data. Third, unlike previous studies that focused on PD with H&Y 1–4 [11], we included PD subjects with H&Y 1–1.5, and both PD and ET patients had limb tremor symptoms, which was more meaningful and challenging for the differentiation between early PD and ET in clinical practice. Fourth, our dataset and final model are highly stable. We added a process at the end to verify the stability of our data and the selected final ensemble model (eDiscussion in the Online Resource).

### Comparison between our model and other methods

Other studies have used EMG or sensors to distinguish between PD and ET. Ghassemi et al. [9] utilized features that were extracted from the tremor component of the hand movement signal obtained from EMG and accelerometer while participants performed standardized upper extremity movement tests to distinguish PD from ET (13 PD and 11 ET) and achieved a LOOCV accuracy of 83%. Although Ghassemi et al.’s study was comparable in accuracy to our study (LOOCV accuracy of 84%), the EMG they utilized is an invasive examination, and when participants had mixed types of tremors, the technology’s applications were limited. Moon et al. [14] utilized gait measures collected from wearable sensors combined with machine learning methods to distinguish PD from ET, which is similar to our research. However, the F1 score of their best model was 0.61. To make the comparison to their study, we calculated the F1 score based on our model and found that the F1 score of our best model was 0.84 for LOOCV and 0.8 for independent test data, suggesting that the accuracy of our model is better than theirs. Additionally, the average disease duration for PD in their study was 8.2 years; therefore, it was not clear if their study could be applied to discriminate between early-stage PD and ET. However, the differential diagnosis of early-stage PD and ET is exactly the clinical challenge, and that is what we have worked on solving.

### Limitations and future study

Our proposed early-stage PD and ET classification model (LOOCV accuracy: 84%, independent test accuracy: 75.8%) is good with proven feasibility and potential to differentiate early-stage PD from ET, but it is not outstanding. Some limitations and future extension need to be considered to make the current study better. First, there are individual differences in gait and postural parameters even in participants who have the same disease. To better represent the population data, future research should include more participants to make the samples more representative and the research more reliable and accurate. Second, in addition to the basic models SVM and RF, other models should also be taken into consideration, which may make the classification framework more accurate. Third, the assessments from our current study were all performed in the clinic with a gait quantitative evaluation system. It is affordable and readily available for the clinic; however, it may not be suitable for personal use at home. In addition to gait parameters obtained from a short and standard test such as the TUG test in the clinic, future work is required to extend this approach to real-world gait assessments with more flexible devices and continuous monitoring and compare it with our current classification framework. Fourth, in addition to gait features, other measurements, such as the measurement of hand tremor variation acquired through tremor signal analysis from rest, postural and kinetic tasks [32] and the measurement of the temperature of participants before and after cold stimuli acquired through the cold stress test [33], could be integrated into our current study. Fifth, although no participants complained about the number of sensors that they needed to wear during the test, sensor number minimization can be considered in our future work and compared with our study to see if we could balance the number of sensors and the overall accuracy of our classification model.

## Conclusion

Our study showed that simple wearable sensors combined with machine learning algorithms and instrumented TUG test had the potential to differentiate early-stage PD from ET.

### Electronic supplementary material

Below is the link to the electronic supplementary material.

Supplementary file1 (DOCX 64 kb)

## Figures and Tables

**Fig. 4:**
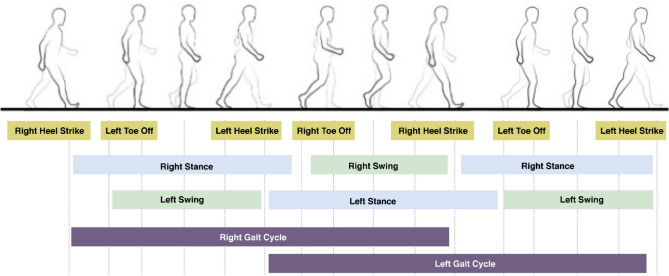
Gait phase of a normal gait cycle

**Fig. 5:**
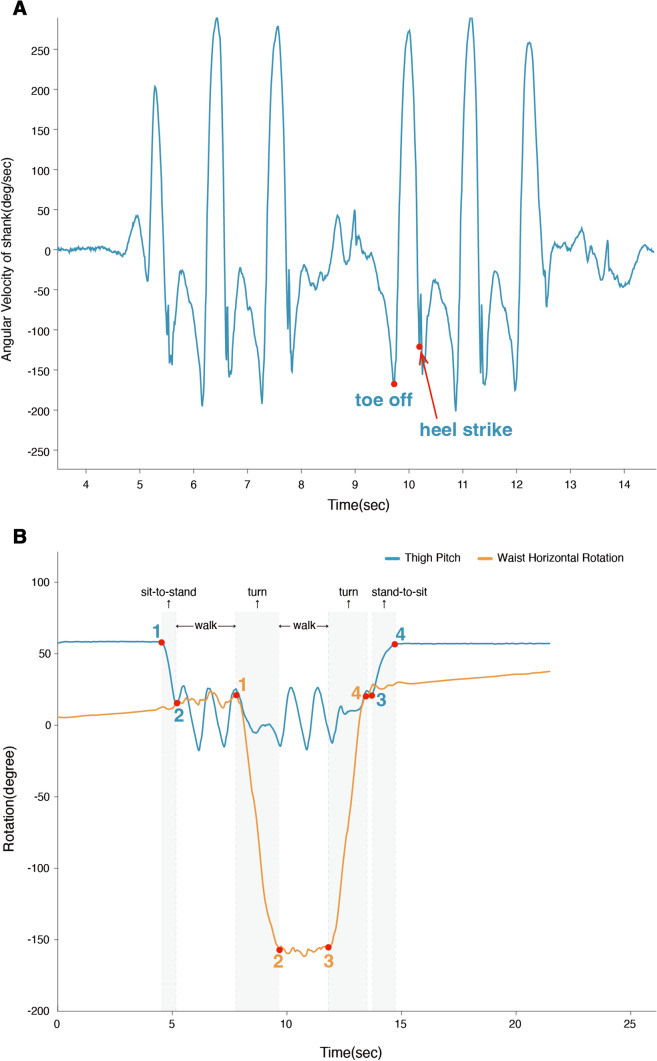
**A** Toe-off and heel-strike detection. **B** TUG test segmentation

## Data Availability

The corresponding author Shengdi Chen had full access to all the data in the study and takes responsibility for the integrity of the data and the accuracy of the data analysis.

## Appendix A

See Table 4.

**Table 4:** Gait and postural transition parameters obtained from the wearable sensors during the TUG test

Gait parameters		
Step length L (cm)	Shank–forward swing max SD (degree)	Trunk–forward sway max SD (degree)
Step length R (cm)	Shank–backward swing max L (degree)	Trunk–backward swaying max (degree)
Step length (cm)	Shank–backward swing max R (degree)	Trunk–backward swaying max SD (degree)
Step length L SD (cm)	Shank–backward swing max (degree)	Trunk–max transverse angular velocity (degree/s)
Step length R SD (cm)	Shank–backward swing max L SD (degree)	Trunk–max transverse angular velocity SD (degree/s)
Step length SD (cm)	Shank–backward swing max R SD (degree)	Trunk–right rotation max (degree)
Gait speed L (m/s)	Shank–backward swing max SD (degree)	Trunk–right rotation max SD (degree )
Gait speed R (m/s)	Shank–max sagittal angular velocity L (degree/s)	Trunk–left rotation max (degree)
Gait speed (m/s)	Shank–max sagittal angular velocity R (degree/s)	Trunk–left rotation Max SD (degree)
Gait speed L SD (m/s)	Shank–max sagittal angular velocity (degree/s)	Lumbar–max coronal angular velocity (degree/s)
Gait speed R SD (m/s)	Shank–max sagittal angular velocity L SD (degree/s)	Lumbar–max coronal angular velocity SD (degree/s)
Gait speed SD (m/s)	Shank–max sagittal angular velocity R SD (degree/s)	Lumbar–right sway max (degree)
Stride length L (cm)	Shank–max sagittal angular velocity SD (degree/s)	Lumbar–right sway max SD (degree)
Stride length R (cm)	Stride velocity asymmetry (%)	Lumbar–left sway max (degree)
Stride length (cm)	Stride velocity asymmetry SD (%)	Lumbar–left sway max SD (degree)
Stride length L SD (cm)	Stride velocity difference (m/s)	Lumbar–max sagittal angular velocity (degree/s)
Stride length R SD (cm)	Stride Velocity Difference SD (m/s)	Lumbar–max sagittal angular velocity SD (degree/s)
Stride length SD (cm)	Stride length asymmetry (%)	Lumbar–forward sway max (degree)
Gait cycle L (s)	Stride length asymmetry SD (%)	Lumbar–forward sway max SD (degree)
Gait cycle R (s)	Stride length difference (cm)	Lumbar–backward swaying max (degree)
Gait cycle (s)	Stride length difference SD (cm)	Lumbar–backward swaying max SD (degree)
Gait cycle L SD (s)	Swing asymmetry (%)	Lumbar–max transverse angular velocity (degree/s)
Gait cycle R SD (s)	Swing asymmetry SD (%)	Lumbar–max transverse angular velocity SD (degree/s)
Gait cycle SD (s)	Swing absolute difference (%)	Lumbar–right rotation max (degree)
Cadence L (step/min)	Swing absolute difference SD (%)	Lumbar–right rotation max SD (degree)
Cadence R (step/min)	Stance asymmetry (%)	Lumbar–left rotation max (degree)
Cadence (step/min)	Stance asymmetry SD (%)	Lumbar–left rotation max SD (degree)
Cadence L SD (step/min)	Stance absolute difference (%)	Arm–max sagittal angular velocity L (degree/s)
Cadence R SD (step/min)	Stance absolute difference SD (%)	Arm–max sagittal angular velocity L SD (degree/s)
Cadence SD (step/min)	Shank–ROM asymmetry (%)	Arm–max sagittal angular velocity R (degree/s)
Double support L (%GCT)	Shank–ROM Asymmetry SD (%)	Arm–max sagittal angular velocity R SD (degree/s)
Double support R (%GCT)	Shank–ROM Absolute Difference (degree)	Arm–Max Sagittal Angular Velocity (degree/s)
Double support (%GCT)	Shank–ROM absolute difference SD (degree)	Arm–max sagittal angular velocity SD (degree/s)
Double support L SD (%GCT)	Shank–asymmetry of max sagittal angular velocity (%)	Arm–forward swing max L (degree)
Double support R SD (%GCT)	Shank–asymmetry of max sagittal angular velocity SD (%)	Arm–forward swing max L SD (degree)
Double support SD (%GCT)	Shank–difference of max sagittal angular velocity (degree/s)	Arm–forward swing max R (degree)
Swing L (%GCT)	Shank–difference of max sagittal angular velocity SD (degree/s)	Arm–forward swing max R SD (degree)
Swing R (%GCT)	Shank–symbolic Symmetry Index (%)	Arm–forward swing max (degree)
Swing (%GCT)	Shank–symbolic symmetry index SD (%)	Arm–forward swing max SD (degree)
Swing L SD (%GCT)	Mean phase difference (%)	Arm–backward swing max L (degree)
Swing R SD (%GCT)	Mean phase difference SD (%)	Arm–backward swing max L SD (degree)
Swing SD (%GCT)	Phase coordination index (%)	Arm–backward swing max R (degree)
Stance L (%GCT)	Phase coordination index SD (%)	Arm–backward swing max R SD (degree)
Stance R (%GCT)	Trunk–max coronal angular velocity (degree/s)	Arm–backward swing max (degree)
Stance (%GCT)	Trunk–max coronal angular velocity SD (degree/s)	Arm–backward swing max SD (degree)
Stance L SD (%GCT)	Trunk–right sway max (degree)	Arm–asymmetry of max sagittal angular velocity (%)
Stance R SD (%GCT)	Trunk–right sway max SD (degree)	Arm–asymmetry of max sagittal angular velocity SD (%)
Stance SD (%GCT)	Trunk–left sway max (degree)	Arm–difference of max sagittal angular velocity (degree/s)
Shank–Forward swing max L (degree)	Trunk–left sway max SD (degree)	Arm–difference of max sagittal angular velocity SD (degree/s)
Shank–forward swing max R (degree)	Trunk–max sagittal angular velocity (degree/s)	Arm–symbolic symmetry index (%)
Shank–forward swing max (degree)	Trunk–max sagittal angular velocity SD (degree/s)	Arm–symbolic symmetry index SD (%)
Shank–forward swing max L SD (degree)	Trunk–forward sway max (degree)	Straight-walking–duration (s)
Shank–forward swing max R SD (degree)		Straight-walking–duration SD (s)
Postural transition parameters		
Sit to stand–duration (s)	Stand to sit–duration (s)	180° Turn–duration (s)
Sit to stand–duration SD (s)	Stand to sit–duration SD (s)	180° Turn–duration SD (s)
Sit to stand–trunk–max sagittal angular velocity (degrees/s)	Stand to sit–trunk–max sagittal angular velocity (degrees/s)	180° Turn–max angular velocity (degree/s)
Sit to stand–trunk–max sagittal angular velocity SD (degrees/s)	Stand to sit–trunk–max sagittal angular velocity SD (degrees/s)	180° Turn–max angular velocity SD (degree/s)
Sit to stand–trunk–min lean angle (degree)	Stand to sit–trunk–min lean angle (degree)	180° Turn–step duration (s)
Sit to stand–trunk–min lean angle SD (degree)	Stand to sit–trunk–min lean angle SD (degree)	180° Turn–step duration SD (s)
Sit to stand -trunk–max lean angle (degree)	Stand to sit–trunk–max lean angle (degree)	180° Turn–mean angular velocity (degree/s)
Sit to stand -trunk–max lean angle SD (degree)	Stand to sit–trunk–max lean angle SD (degree)	180° Turn–mean angular velocity SD (degree/s)
		180° Turn–steps (#)
		180° Turn–steps SD (#)

*GCT* gait cycle time; *SD* standard deviation

## Appendix B

See Table 5.

**Table 5:** LOOCV performance and weights of four different components in all model and feature group combinations

Model	Feature Group (FG)	Component	Val ACC (%)	Val kappa	Val sensitivity (%)	Val specificity (%)	Val AUC	Weight
**SVM**	I	Straight walk	78.6	0.573	79.7	77.6	0.821	0.457
		Turning	67.9	0.354	51.6	83.6	0.692	0.202
		Standing	60.3	0.213	79.7	41.8	0.700	0.314
		Sitting	61.1	0.226	75.0	47.8	0.739	0.027
	II	Straight walk	76.3	0.529	87.5	65.7	0.807	0.392
		Turning	66.4	0.332	78.1	55.2	0.715	0.217
		Standing	76.3	0.531	95.3	58.2	0.920	0.354
		Sitting	58.0	0.170	84.4	32.8	0.615	0.037
	**III**	**Straight walk**	**78.6**	**0.572**	**76.6**	**80.6**	**0.850**	**0.435**
		**Turning**	**67.9**	**0.356**	**57.8**	**77.6**	**0.702**	**0.188**
		**Standing**	**76.3**	**0.531**	**95.3**	**58.2**	**0.920**	**0.352**
		**Sitting**	**61.1**	**0.226**	**75.0**	**47.8**	**0.739**	**0.026**
RF	I	Straight walk	73.3	0.465	72.0	75.0	0.780	0.723
		Turning	64.9	0.295	56.0	73.0	0.658	0.016
		Standing	67.2	0.342	62.0	72.0	0.698	0.163
		Sitting	59.5	0.187	48.0	70.0	0.635	0.098
	II	Straight walk	79.4	0.586	70.0	88.0	0.836	0.490
		Turning	61.8	0.235	58.0	66.0	0.677	0.395
		Standing	74.0	0.482	81.0	67.0	0.689	0.104
		Sitting	58.0	0.155	44.0	72.0	0.540	0.011
	III	Straight walk	71.8	0.433	64.0	79.0	0.811	0.489
		Turning	64.9	0.295	58.0	72.0	0.658	0.299
		Standing	74.0	0.482	81.0%	67.0%	0.689	0.203
		Sitting	59.5	0.187	48.0%	70.0%	0.635	0.008

The significance of bold were used to highlight the best performance among all the others in Table 5

## Appendix C: Introduction of the gait cycles and segmentation of the TUG

According to the wearing mode of each sensor, the IMU coordinate system was converted to the human body coordinate system. Then, combined with the accelerometer and gyroscope of the IMU, and after complementary filtering, the attitude information of each sensor was calculated, including the pitch angle, roll angle and horizontal rotation, and then the raw gyroscope data of the IMU were utilized to identify the moment when the toe-off (TO) and heel-strike (HS) events occurred and the attitude information was utilized to identify the moment when the standing, straight walk, turning and sitting events occurred.

Gait cycles were detected by HS and TO events (Appendix C Fig. 4). The right gait cycle begins from the right HS, then right TO, and then the right HS. The left gait cycle begins from the left HS, then the left TO, and then the left HS. Detailed definitions of gait phases are listed below—

1. Right (Left) Stance: In a right (left) gait cycle, the percentage of time from the right (left) HS to right (left) TO.
2. Right (Left) Swing: In a right (left) gait cycle, the percentage of time from the right (left) TO to right (left) HS.

Salarian et al*.* [34] found that the gyroscope signal of the shank was more sensitive in detecting TO and HS. The first minimum value before and after each peak angular velocity of the shank was considered the time when the TO and HS events occurred. In the same way, we detected TO and HS events during walking, as shown in Appendix C Fig. 5A, and combined them into a gait cycle.

The TUG test was divided into standing, straight walk, turning and sitting. Standing and sitting were recognized using a thigh sensor. As shown in Appendix C Fig. 5B, the first and second spots in the thigh pitch angle curve (blue line) indicate the start and end of standing, and the third and fourth spots indicate the start and end of sitting. The change in the waist horizontal rotation angle can identify the start and end moments of the two turns. The first and second spots of the waist horizontal rotation angle curve (orange line) indicate the start and end of the first turn, and the third and fourth spots indicate the start and end of the second turn. It can be seen that the first straight walk section is from the end of standing up to the beginning of the first turn, and the second straight walk section is from the end of the first turn to the start of the second turn, thus comprising the phase divisions in the TUG test. During the straight walk section, individual gait cycles were detected and analyzed across the whole trial, and the average values of the 158 gait parameters (Appendix A Table 4) were investigated. During the turning, standing, and sitting components, 10, 8 and 8 postural transition parameters (Appendix A Table 4) were investigated, respectively. Feature construction (eMethods in the Online Resource) was then performed to address the gait and postural transition parameters.

## Appendix D: Feature selection methods

Method 1: The Mann‒Whitney U test was performed to investigate which features differed significantly between PD and ET, and we called this feature combination Feature Group I (FG I).

Method 2: Spearman's rank correlation test was performed for every pair of the above significant features to remove features with high correlations (Set threshold *ρ* = 0.6) following the High-correlation Features Removal Rule (eMethods in the Online Resource). We called this feature combination, which was kept as Feature Group II (FG II).

Method 3: Fivefold cross-validation ROC area under the curve (AUC) of the above significant features obtained from Method 1 was computed individually. Then, considering that (1) the AUC values of our features are all lower than 0.7 and (2) AUC ≥ 0.6 represents that it has the power to be at least not fail for a diagnostic test, which we found from one previous study [35], we extracted features that had AUC ≥ 0.6 and called this feature combination Feature Group III (FG III).

## Appendix E: Weighted average ensemble classification model construction

This ensemble classification model construction included 18 steps:

1. The entire TUG test was separated into four components: straight walk, turning, standing, and sitting.
2. The response variable of each of the four component models was the diagnosis result – whether the subject was PD or ET (we defined PD as 1, ET as 0). Predictors were considered among the three-feature selection method results FG I, FG II, and FG III.
3. Four components, namely, straight walk, turning, standing, and sitting, were trained separately using the training data with features from the corresponding components through LOOCV. Two types of basic models were tried here: support vector machine (SVM) and random forest (RF) models. For example, for the straight walk component, we only extracted the features related to straight walk from FG I and then utilized these features as input features to train the basic SVM and RF models ordinally through LOOCV to fine-tune the model parameters to obtain the best SVM model and best RF model for the straight walk component. Turning, sitting, and standing components followed the same process as the Straight Walk component. Therefore, there were 6 model and feature group combinations in total: FG I + basic model SVM, FG I + basic model RF, FG II + basic model SVM, FG II + basic model RF, FG III + basic model SVM, and FG III + basic model RF.
4. For each of the feature groups and basic model combinations mentioned in Step 3, we repeated Step 5 to Step 13.
5. The predicted probabilities of the disease being PD for each subject in the training data group obtained from each of the four component models were calculated and saved. In this case, each subject in the training data group should have four probabilities of being a PD, which were called predicted scores, corresponding to the four different components. The 4 predicted scores were noted as $P_{{{\text{straight}}\_{\text{walk}}\_{\text{train}}}}$, $P_{{{\text{turning}}\_{\text{train}}}}$, $P_{{{\text{standing}}\_{\text{train}}}}$ and $P_{{{\text{sitting}}\_{\text{train}}}}$.
6. We combined the original response variable (diagnosis result) in the training data and 4 columns of the predicted probabilities above as a new dataset. The original response variable was also the response variable in the new dataset, and the other 4 columns of the predicted probabilities were used as the predictors.
7. Logistic regression was run on this new dataset with a fivefold cross-validation method to fine-tune the model parameters to obtain the best logistic regression model.
8. The captured 4 coefficients (ignoring the intercept) derived from the above logistic regression analysis corresponded to the four different components. These 4 coefficients were annotated as ${\text{Coefficient}}_{{{\text{straight}}\_{\text{walk}}}}$,${\text{Coefficient}}_{{{\text{turning}}}}$,${\text{Coefficient}}_{{{\text{standing}}}}$, and ${\text{Coefficient}}_{{{\text{sitting}}}}$ respectively.
9. Four linear weights were calculated based on the coefficients obtained in Step 8:
  1 $${\text{Weight}}_{{{\text{straight}}\_{\text{walk}}}} = \frac{{|{\text{Coefficient}}_{{{\text{straight}}\_{\text{walk}}}} |}}{{|{\text{Coefficient}}_{{{\text{straight}}\_{\text{walk}}}} | + |{\text{Coefficient}}_{{{\text{turning}}}} | + |{\text{Coefficient}}_{{{\text{standing}}}} | + |{\text{Coefficient}}_{{{\text{sitting}}}} |}}$$
  2 $${\text{Weight}}_{{{\text{turning}}}} = \frac{{|{\text{Coefficient}}_{{{\text{turning}}}} |}}{{|{\text{Coefficient}}_{{{\text{straight}}\_{\text{walk}}}} | + |{\text{Coefficient}}_{{{\text{turning}}}} | + |{\text{Coefficient}}_{{{\text{standing}}}} | + |{\text{Coefficient}}_{{{\text{sitting}}}} |}}$$
  3 $${\text{Weight}}_{{{\text{standing}}}} = \frac{{|{\text{Coefficient}}_{{{\text{standing}}}} |}}{{\left| {{\text{Coefficient}}_{{{\text{straight}}\_{\text{walk}}}} } \right| + \left| {{\text{Coefficient}}_{{{\text{turning}}}} } \right| + \left| {{\text{Coefficient}}_{{{\text{standing}}}} } \right| + \left| {{\text{Coefficient}}_{{{\text{sitting}}}} } \right|}}$$
  4 $${\text{Weight}}_{{{\text{sitting}}}} = \frac{{\left| {{\text{Coefficient}}_{{{\text{sitting}}}} } \right|}}{{\left| {{\text{Coefficient}}_{{{\text{straight}}\_{\text{walk}}}} } \right| + \left| {{\text{Coefficient}}_{{{\text{turning}}}} } \right| + \left| {{\text{Coefficient}}_{{{\text{standing}}}} } \right| + \left| {{\text{Coefficient}}_{{{\text{sitting}}}} } \right|}}$$
10. The calculated ensemble learning prediction probability score of each subject in the training data, $P\_{\text{val}}$ was obtained by multiplying the weights obtained from Step 9 with the predicted scores from the training data obtained from the four different component models in Step 5:
  5 $$P\_{\text{val}}\, =\, {\text{Weight}}_{{{\text{straight}}\_{\text{walk}}}} \times P_{{{\text{straight}}\_{\text{walk}}\_{\text{train}}}} + {\text{Weight}}_{{{\text{turning}}}} \times P_{{{\text{turning}}\_{\text{train}}}} + {\text{Weight}}_{{{\text{standing}}}} \times P_{{{\text{standing}}\_{\text{train}}}} + {\text{Weight}}_{{{\text{sitting}}}} \times P_{{{\text{sitting}}\_{\text{train}}}}$$
11. If the $P\_{\text{val}}$ was > 0.5, the subject would be classified as having PD using our model; otherwise, they would be classified as having ET.
12. The confusion matrix was constructed using the predicted label obtained from Step 11 and the true label (true diagnosis result) from the subjects in the training data.
13. The cross-validation performance (accuracy, kappa, sensitivity, and specificity) of this weighted average ensemble classification model was calculated based on the confusion matrix constructed in Step 12, and the cross-validation performance (AUC) was calculated using the predicted value, $P\_{\text{val}}$ obtained in Step 10 and the true labels from the training data.
14. The weighted average ensemble classification model, which had the best validation performance among the models, was selected as our final model.
15. Predicted on test data using four trained models (straight walk, turning, standing, and sitting) from the best weighted average ensemble classification model separately and obtain the corresponding predicted probabilities of being a PD corresponding to the four different components for each subject in the test data. The 4 predicted probabilities were noted as $P_{{{\text{straight}}\_{\text{walk}}}}$, $P_{{{\text{turning}}}}$, $P_{{{\text{standing}}}}$ and $P_{{{\text{sitting}}}}$.
16. The calculated ensemble learning prediction probability score of each subject in the test data group $P$ was obtained by multiplying weights obtained from Step 9 with test data predicted scores obtained from four different component models in Step 15:
  6 $$P\, =\, {\text{Weight}}_{{{\text{straight}}\_{\text{walk}}}} \times P_{{{\text{straight}}\_{\text{walk}}}} + {\text{Weight}}_{{{\text{turning}}}} \times P_{{{\text{turning}}}} + {\text{Weight}}_{{{\text{standing}}}} \times P_{{{\text{standing}}}} + {\text{Weight}}_{{{\text{sitting}}}} \times P_{{{\text{sitting}}}}$$
17. If $P$ was > 0.5, the subject in the test data would be classified as having PD using our model; otherwise, they would be classified as having ET.
18. The test data performance (AUC) was calculated using the predicted value, $P$, obtained in Step 16 and the true labels of the test data; the test data performance (accuracy, kappa, sensitivity, specificity) was calculated using the predicted labels obtained in Step 17 and the true labels of the test data.

## Appendix F: Example of the ensemble learning prediction probability score calculation

For example, the ensemble learning prediction probability score $P\_val$ of each subject in the training data group obtained from the weighted average ensemble classification models with the basic model SVM and FG III was calculated as follows:

5 $$P\_{\text{val}} = {\text{Weight}}_{{{\text{straight}}\_{\text{walk}}}} \times P_{{{\text{straight}}\_{\text{walk}}\_{\text{train}}}} + {\text{Weight}}_{{{\text{turning}}}} \times P_{{{\text{turning}}\_{\text{train}}}} + {\text{Weight}}_{{{\text{standing}}}} \times P_{{{\text{standing}}\_{\text{train}}}} + {\text{Weight}}_{{{\text{sitting}}}} \times P_{{{\text{sitting}}\_{\text{train}}}}$$

where ${\text{Weight}}_{{{\text{straight}\_\text{walk}}}}$ = 0.435, ${\text{Weight}}_{{{\text{turning}}}}$ = 0.188,${\text{Weight}}_{{{\text{standing}}}}$ = 0.352, ${\text{Weight}}_{{{\text{sitting}}}}$ = 0.026 and ${\text{P}}_{{{\text{straight\_walk\_train}}}}$, ${\text{P}}_{{{\text{turning\_train}}}}$, ${\text{P}}_{{{\text{standing\_train}}}}$, ${\text{P}}_{{{\text{sitting\_train}}}}$ are the predicted probabilities of the disease being PD for each subject in the training data group obtained from each of the four component models in SVM with FG III.
