# Supplementary material for: Wearable sensor-based gait analysis to discriminate early Parkinson’s disease from essential tremor
Source: J Neurol. 2023 Feb 1;270(4):2283–301. doi: 10.1007/s00415-023-11577-6 (PMC10025195; doi:10.1007/s00415-023-11577-6)
Supplement: Supplementary file 1 — Supplementary file1 (DOCX 64 kb) [file 415_2023_11577_MOESM1_ESM.docx]

**Supplementary Materials for**

**Wearable Sensor-based Gait Analysis to Discriminate Early Parkinson's Disease from Essential Tremor**

Shinuan Lin, MSc^a,c*^, Chao Gao, MD, PhD^b*^, Hongxia Li, MD, PhD^b*^, Pei Huang, MD, PhD^b^, Yun Ling, BSc^a,c^, Zhonglue Chen, MSc^a,c^, Kang Ren, PhD^a,c^, and Shengdi Chen, MD, PhD ^b+^

^a^ GYENNO SCIENCE CO., LTD., Shenzhen, China, 518000

^b^ Department of Neurology，Ruijin Hospital, Shanghai Jiao Tong University School of Medicine, 197 Ruijin Er Road, Shanghai, China, 200025

^c^ HUST – GYENNO CNS Intelligent Digital Medicine Technology Center, Wuhan, China, 430074

^+^**Correspondence to:**

Shengdi Chen, Department of Neurology, Ruijin Hospital, Shanghai Jiaotong University School of Medicine, 197 Ruijin Er Road, Shanghai, P.R. China; Tel.: +86+21+6445-4473; Email: chensd@rjh.com.cn.

^*^These authors share the first authorship of this article.

This file includes:

eMethods

eDiscussion

eFig. 1 Feature Overview

eFig. 2 ROC curves for three different data splits

eTable 1 Features kept after Spearman's rank correlation test

**eMethods**

**Feature Construction**

Within the 158 gait parameters, for parameters ***Arm - Forward Swing Max L*** and ***Arm - Backward Swing Max L***, we defined a new feature ***Arm - Swing Range of Motion (ROM) L*** which was the range between ***Arm - Forward Swing Max L*** and ***Arm - Backward Swing Max L*** to replace them. Same process was performed for ***Arm - Forward Swing Max R*** and ***Arm - Backward Swing Max R***, ***Shank - Forward Swing Max L*** and ***Shank - Backward Swing Max L***, ***Shank - Forward Swing Max R*** and ***Shank - Backward Swing Max R***. Thus, these original 8 gait parameters were replaced by two new pairs of features ***Arm - Swing ROM L*** and ***Arm - Swing ROM R***, ***Shank - Swing ROM L*** and ***Shank - Swing ROM R***, which resulted in 154 gait parameters [158 (original) – 8 (original Arm (Shank) – Backward (Forward) Swing Max L(R)) + 4 (two new pairs of Swing ROM)] in total. Among these 154 gait parameters, there were 34 right-sided parameters and 34 left-sided parameters. These 34 pair parameters have same definition except for different side. In general, the human body appears symmetrical with regard to the left-right axis. However, unilateral disease, dominant side (left/right), and habit may cause asymmetry on the left and right sides of human. In order to reduce the impact of this asymmetry on the overall analysis, for each pair of right-sided and left-side parameters, the following construction methods were performed: (1) New feature 1: Max (left feature, right feature); (2) New feature 2: Min (left feature, right feature), (3) New feature 3: abs (left feature - right feature). These three new features replaced the pair of original right-sided and left-sided parameters. Therefore, there were 188 gait features [154 (obtained above) – 34*2 (original pairs of left and right features) + 34*3 (three new features for each pair of left and right features)] instead of 158 gait parameters in total after feature construction. For example, after feature construction, the original right-sided and left-sided parameter ***Step Length L*** and ***Step Length R*** were replaced by these three new features: ***Step Length (Max)***, ***Step Length (Min)***, and ***Step Length (abs)***. Max noted for maximum value between ***Step Length L*** and ***Step Length R***, Min noted for minimum value between ***Step Length L*** and ***Step Length R***, abs noted for the absolute value of the difference between ***Step Length L*** and ***Step Length R***. The feature overview is shown in eFig. 1.

**High-correlation Features Removal Rule**

Spearman's correlation was used as a more conservative estimation method compared to Pearson's, although their results were similar in general. Specifically, the following rule was followed when the features with high spearman correlations were removed. For the two features which had high correlation (ρ>=0.6), the Mean Absolute Correlations for these two features were calculated respectively, the one had high Mean Absolute Correlation would be removed. The Mean Absolute Correlation for each remaining feature would be re-calculated each time after feature removal. As noted, Mean Absolute Correlation was the average of the absolute correlations between a specific feature and other features.

**eDiscussions**

**High Stability of Used Data & Model**

As we mentioned in the Methods section, we randomly split our entire dataset into training and test data at the beginning. The red line in eFigure 2 represented the LOOCV ROC curve of our study. We set another two random seeds to randomly split our entire dataset into training data (80%) and test data (20%), and repeat all the analysis and processes twice. The blue line and green line in eFigure 2 showed the corresponding LOOCV ROC curves for the other two random seeds respectively. We found that all the three LOOCV AUCs were between 0.86 and 0.91, which were stable and acceptable.

**
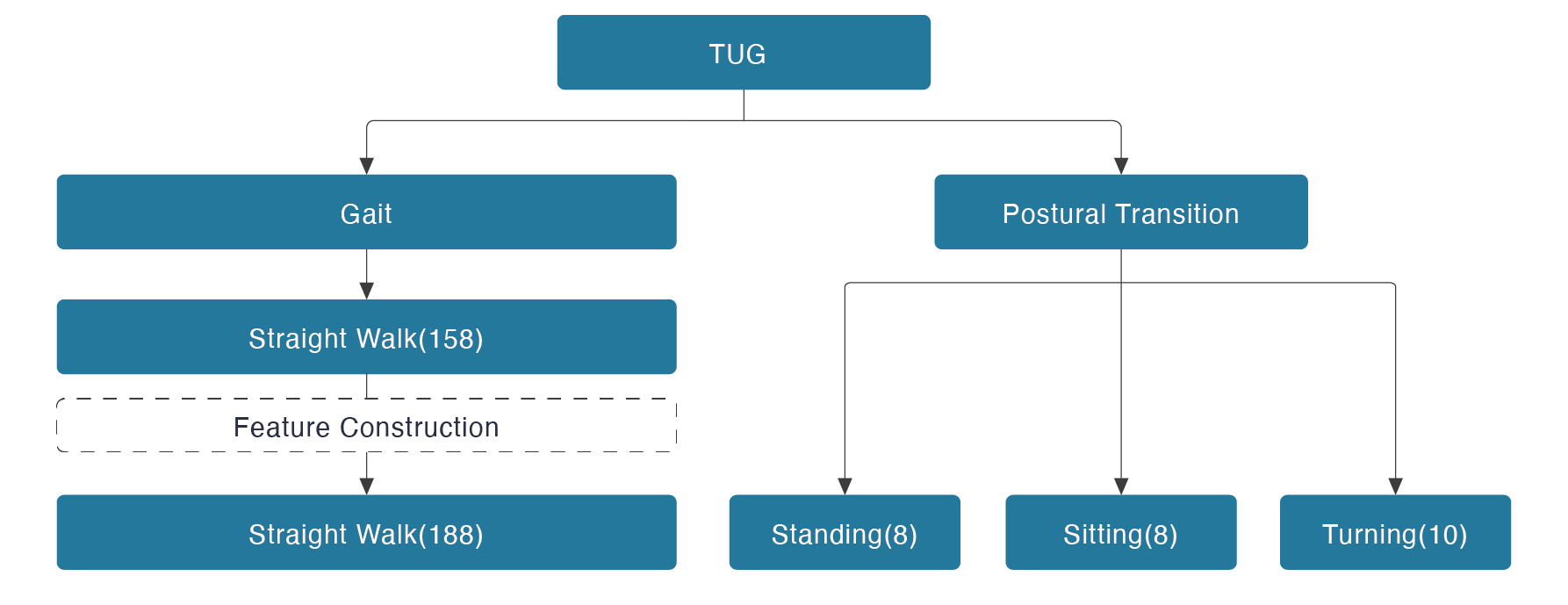
eFig. 1 Feature Overview**

**
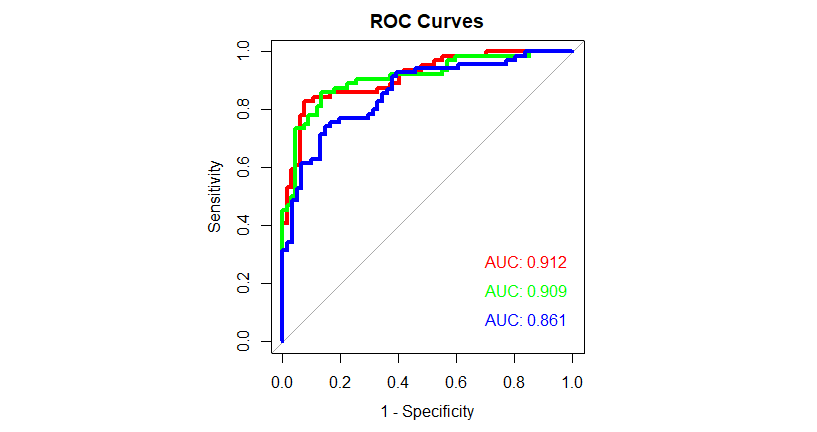
**

**eFig. 2 ROC curves for three different data splits**

**eTable 1 Features kept after Spearman's rank correlation test**

| **Feature name** | **ET Median (IQR)** | **PD Median (IQR)** |
| --- | --- | --- |
| Trunk - Max Sagittal Angular Velocity (degree/s) | 35.619 (10.901) | 33.076 (13.583) |
| Trunk - Forward Sway Max (degree) | 20.845 (11.795) | 15.708 (14.064) |
| Lumbar - Max Sagittal Angular Velocity (degree/s) | 48.211 (27.541) | 53.93 (28.267) |
| Arm - Symbolic Symmetry Index (%) | 34.29 (8.115) | 39.762 (6.697) |
| Arm - Symbolic Symmetry Index SD (%) | 9.567 (2.996) | 8.146 (2.563) |
| Sit To Stand - Trunk - Max Sagittal Angular Velocity (degrees/s) | 89.167 (30.547) | 72.291 (25.728) |
| Stand To Sit - Trunk - Max Sagittal Angular Velocity (degrees/s) | 77.048 (35.079) | 64.461 (31.947) |
| 180° Turn - Max Angular Velocity (degree/s) | 160.701 (40.701) | 139.817 (41.601) |
| 180° Turn - Mean Angular Velocity SD (degree/s) | 2.422 (3.822) | 3.919 (6.19) |
| 180° Turn - Steps (#) | 2 (1) | 2 (0.625) |
| Arm - Swing ROM (Min) (degree) | 27.349 (18.67) | 16.368 (19.533) |
| Arm - Swing ROM (abs) (degree) | 6.964 (10.882) | 14.281 (17.18) |
| Step Length SD (abs) (cm) | 1.26 (2.041) | 2 (2.78) |
| Gait Speed SD (Min) (m/s) | 0.056 (0.035) | 0.065 (0.039) |
| Stride Length SD (abs) (cm) | 2.509 (3.005) | 3.955 (5.03) |
| Cadence (abs) (step/min) | 4.503 (5.902) | 6.002 (10.24) |
| Cadence SD (Max) (step/min) | 5.932 (2.147) | 6.564 (3.153) |
| Double Support (abs) (%GCT) | 0.741 (0.934) | 1.19 (1.198) |
| Double Support SD (Max) (%GCT) | 3.086 (1.468) | 3.679 (1.981) |
| Shank - Forward Swing Max SD (Max) (degree) | 2.54 (1.095) | 2.903 (1.159) |
| Shank - Forward Swing Max SD (Min) (degree) | 1.557 (0.758) | 1.885 (1.088) |
| Shank - Max Sagittal Angular Velocity SD (abs) (degree/s) | 7.171 (6.877) | 11.32 (15.578) |
| Trunk - Sway Max (abs) (degree) | 4.717 (2.511) | 3.878 (2.553) |
| Trunk - Sway Max SD (Max) (degree) | 0.96 (0.461) | 0.861 (0.415) |
| Trunk - Rotation Max SD (abs) (degree) | 0.544 (0.772) | 1.076 (1.642) |
| Lumbar - Sway Max (Max) (degree) | 3.873 (2.732) | 1.986 (3.042) |
| Lumbar - Sway Max (abs) (degree) | 5.375 (2.68) | 4.423 (2.583) |
| Lumbar - Rotation Max SD (abs) (degree) | 0.582 (1.176) | 0.858 (1.845) |
| Arm - Max Sagittal Angular Velocity SD (abs) (degree/s) | 6.827 (12.898) | 13.458 (17.945) |
| Shank - Forward Swing Max SD (degree) | 2.781 (1.659) | 3.485 (1.843) |
| Stride Length Difference SD (cm) | 2.533 (3.017) | 3.344 (3.03) |
| Shank - ROM Absolute Difference SD (degree) | 1.852 (1.565) | 2.488 (1.944) |
| Shank - Difference of Max Sagittal Angular Velocity (degree/s) | 22.622 (20.907) | 29.079 (27.511) |
| Arm - Max Sagittal Angular Velocity SD (degree/s) | 31.25 (18.404) | 37.095 (31.581) |

Abbreviations: ET, essential tremor; PD, Parkinson's disease; GCT, Gait Cycle Time; ROM, Range of Motion; Max, Maximum value between the pair of left-sided and right-sided parameters; Min, Minimum value between the pair of left-sided and right-sided parameters; abs, The absolute value of the difference between the left-sided parameter and right-sided parameter in the pair.
